# DNA Methylation Profiling of the Fibrinogen Gene Landscape in Human Cells and during Mouse and Zebrafish Development

**DOI:** 10.1371/journal.pone.0073089

**Published:** 2013-08-21

**Authors:** Silja Vorjohann, Jean-Luc Pitetti, Serge Nef, Carmen Gonelle-Gispert, Leo Buhler, Richard J. Fish, Marguerite Neerman-Arbez

**Affiliations:** 1 Department of Genetic Medicine and Development, University of Geneva Medical Centre, Geneva, Switzerland; 2 Institute of Genetics and Genomics in Geneva (iGE3), Geneva, Switzerland; 3 Surgical Research Unit, Department of Surgery, University Hospital, Geneva, Geneva, Switzerland; 4 Division of Angiology and Hemostasis, University Hospital, Geneva, Switzerland; National Institutes of Health, United States of America

## Abstract

The fibrinogen genes *FGA*, *FGB* and *FGG* show coordinated expression in hepatocytes. Understanding the underlying transcriptional regulation may elucidate how their tissue-specific expression is maintained and explain the high variability in fibrinogen blood levels. DNA methylation of CpG-poor gene promoters is dynamic with low methylation correlating with tissue-specific gene expression but its direct effect on gene regulation as well as implications of non-promoter CpG methylation are not clear. Here we compared methylation of CpG sites throughout the fibrinogen gene cluster in human cells and mouse and zebrafish tissues. We observed low DNA methylation of the CpG-poor fibrinogen promoters and of additional regulatory elements (the liver enhancers CNC12 and PFE2) in fibrinogen-expressing samples. In a gene reporter assay, CpG-methylation in the *FGA* promoter reduced promoter activity, suggesting a repressive function for DNA methylation in the fibrinogen locus. In mouse and zebrafish livers we measured reductions in DNA methylation around fibrinogen genes during development that were preceded by increased fibrinogen expression and tri-methylation of Histone3 lysine4 (H3K4me3) in fibrinogen promoters. Our data support a model where changes in hepatic transcription factor expression and histone modification provide the switch for increased fibrinogen gene expression in the developing liver which is followed by reduction of CpG methylation.

## Introduction

Fibrinogen is the soluble precursor of fibrin, the central blood clotting agent in wound healing. Two sets of three polypeptide chains Bß, Aα and γ form the hexameric fibrinogen. The chains are encoded by the genes *FGB, FGA* and *FGG*, which are clustered on a 50 kb region with conservation across all terrestrial vertebrates. Fibrinogen gene expression is limited to hepatocytes and several studies indicate a coordinated regulatory mechanism [1]. Transcription factor binding sites have been analyzed in all three promoter regions with hepatocyte nuclear factor-1 (HNF-1) and CCAAT-box/enhancer-binding protein (C/EBP) being key contributors to basal fibrinogen expression [2], [3]. Identifying additional regulators contributing to the coordinated liver-specific expression of the fibrinogen genes may provide explanations for the variability of fibrinogen blood levels [4]. Smoking, pregnancy and inflammation are among the factors that increase fibrinogen blood level, which is a risk factor for cardiovascular diseases when exceeding the normal range of 1.5 to 3.5 g/L [5]. With a heritability estimated between 20–50%, genetic background also influences variation in fibrinogen plasma levels [6].

DNA methylation may contribute to the regulation of fibrinogen gene expression since it is implicated in cell-specific silencing of gene expression [7], seems to be influenced by environmental and physiological factors [8] and varies even among monozygotic twins [9,10]. DNA methylation in vertebrates mainly occurs on cytosines in a CpG-context. While the majority of the vertebrate genome displays a low CpG-frequency, some regions contain clusters of CpG-rich regions known as CpG-islands [11,12]. Since they are associated with transcriptional start sites and are present in around 70% of all promoters [13] most of the studies evaluating the role of DNA methylation in gene regulation have focused on CpG-islands. These regions are most often unmethylated independent of their expression status [14], and if methylation occurs it is implicated in long-term silencing during X-chromosome inactivation, imprinting, and silencing of germ-cell-specific genes [7,15]. Much less is known about the regulatory function of non-CpG-island promoter (intermediate- and poor CpG- promoter) methylation, even though it seems to be more dynamic [16,17]. Genome-wide studies indicate that non-CpG-island promoters are present in tissue-specific genes and show an inverse relationship between DNA methylation and active gene expression [17]. Additionally, methylation of CpG-poor promoters leads to silencing of tissue-specific genes *in vitro* [18]. However, it is not clear if DNA methylation of non-CpG-island promoters is a cause or a consequence of gene silencing and if loss of DNA methylation plays a role in activating gene expression [12].

Histone modifications are thought to work co-ordinately with DNA methylation in setting up a permissive or closed chromatin structure to regulate gene expression. Trimethylation of lysine 4 in histone 3 (H3K4me3) marks active promoters while acetylation of lysine 27 in histone 3 (H3K27ac) appears to mark active regulatory elements. Both modifications correlate with low DNA methylation levels [19]. DNA-methyltransferases and histone-modifying enzymes connect both epigenetic marks, e.g. DNA-methyltransferase DNMT3L preferentially interacts with unmodified H3K4 (H3K4m0) [20].

Here, we investigated the role of DNA methylation in the regulation of fibrinogen gene expression. We analyzed the methylation status of the whole fibrinogen locus in human cells and in mouse and zebrafish tissues in relation to changing fibrinogen expression levels during development. We found that loss of DNA methylation across the fibrinogen locus during liver development is preceded by an increase in fibrinogen gene expression and by increased H3K4me3 on all three fibrinogen gene promoters.

## Materials and Methods

### Tissue samples

Authorization for human hepatocyte isolation from surgical liver biopsies was obtained from the Institutional Ethics Committee of the Department of Surgery. This study was conducted under experimental protocols and consent procedures approved by the Ethical Committee of the Geneva University Medical School. All patients gave written consent. Animal studies were approved by the Ethical Committee for Animal Experimentation of the Geneva University Medical School and the Canton of Geneva Animal Experimentation Veterinary authority.

Embryos from C57BL/6 mice (Charles Rivers) were staged by designating noon of the day on which the mating plug was detected as embryonic day (E) 0.5 and then collected at E12.5 and E17.5 as described [21]. Embryos and 8 week old C57BL/6 mice were used for liver and heart dissections (tissues directly stored at -80°C). AB strain zebrafish were kept in stable aquarium conditions. Embryos were obtained by natural matings and raised as described previously [22]. Tissue dissection was performed on 3 month-old females (liver and fin or trunk) and 8 day embryos (liver and trunk) using a Leica S6E stereozoom microscope. Liver samples may contain traces of other tissues i.e. pancreas. Trunks are a heterogeneous control sample consisting of trunk tissue. Thirty embryonic livers or trunks were pooled per RNA- or DNA-isolation.

### Cells

HEK293T embryonic kidney cells, HuH7 hepatoma cells and HepG2 hepatoma cells were cultured as described [23]. IL-6 (eBioscience, Vienna, Austria) was added to the cells at a final concentration of 100 ng/ml. Human hepatocytes were isolated from surgical liver biopsies of patients undergoing segmental hepatectomies. Human liver tissue (between 15–30g) was surgically removed during liver resections for benign or malignant diseases by surgeons. Human hepatocyte isolation was performed using a two-step collagenase perfusion method as described [24]. Briefly, resected liver was perfused with Ca^2+^-free buﬀer washing solution (10 ml/min, during 20 min) followed by collagenase solution (type I, 1 mg/ml at 5 ml/min, during 15 min; Sigma, St Louis, MO, USA). Hepatocytes were released by mincing and shaking the liver in a Petri dish containing cold RPMI (Invitrogen) and 10% fetal bovine serum (FBS; Invitrogen). The cell suspension was ﬁltered through a sterile 100 µm nylon mesh into a beaker placed on ice and then transported to the Surgical Research Unit, University of Geneva Medical School in Geneva, where human hepatocytes were puriﬁed by sedimentation on 30% Percoll isodensity solution (Pharmacia Biotech AB, Uppsala, Sweden), as previously described [25]. Viability was assessed by trypan blue exclusion. Cells were cultured in DMEM/F12 medium (Invitrogen, Basel, Switzerland) containing 2% Fetal bovine serum (Invitrogen), 1×10^−6^ mol/l dexamethasone (Sigma-Aldrich GmbH, Basel, Switzerland), 1×10^−8^ mol/l 3,3’ triiodo-L-thyronine, 1×10^−8^ mol/l human insulin (Huminsulin, Lilly France S.A.S, Strasbourg, France), 5 µg/ml apotransferrin (Sigma-Aldrich GmbH), 15×10^−3^ mol/l Hepes. Additionally, one biological replicate of primary human hepatocytes was purchased and grown in HCM bulletkit medium, both from Lonza (Walkersville, MD, USA). Human umbilical vein endothelial cells (HUVECs) were kindly provided by Dr. Sylvie Dunoyer-Geindre (Department of Internal Medicine, University Hospitals, Geneva).

### Bisulfite DNA methylation analysis

Genomic DNA from cell lines and human primary cells was isolated using DNeasy Blood & Tissue kit (QIAGEN, Hilden, Germany). Genomic DNA from animal tissues was isolated using proteinase/phenol extraction. Bisulfite DNA conversion was performed using the EpiTect Bisulfite Kit (QIAGEN, Hilden, Germany). PCRs on bisulfite treated DNA were performed with primers listed in Table S1 using the KAPA2G Robust HotStart ReadyMix (Kapa Bioystems, Boston, Massachusetts, United States). Methylation of individual CpG sites was detected by direct sequencing using T-Tracking (QIAGEN, Hilden, Germany) [26].

### Quantitative RT-PCR (qPCR)

Total RNA from cells and animal tissues was isolated using Trizol (Invitrogen). cDNA was made from 1µg or 400ng RNA using SuperscriptII (Invitrogen) and poly-dT primers. Triplicate qPCR reactions were made for each sample using SYBR green master mix (Applied Biosystems, Foster City, CA, USA). Primer sequences are listed in Table S1. Relative expression levels in human cells were calculated using the ΔΔCT method [27]. Threshold cycle (C_T_) values were normalized to the geometric mean C_T_ of three references genes: elongation factor 1 alpha (*EF1A*), beta-2-microglobulin (*B2M*) and TATA box binding protein (*TBP*). Fibrinogen gene expression was calculated for mouse and zebrafish samples using cloned cDNA standard curves as a reference. Mouse cDNA clones were made using pCRII TOPO (Invitrogen), zebrafish cDNA clones were described previously [22]. Gene expression was calculated as: quantity = 10(C_T_-b)/m, where b is the standard curve y-intercept and m the linear regression slope. Expression is described as cDNA copies per µg RNA, 10fg of the cDNA clones was taken as 1500 copies.

### Western blotting

HuH7 and HepG2 cells were incubated with or without IL-6 for 24 h, washed with phosphate buffered saline (PBS) and incubated for an additional 24 h in media without serum. Conditioned media was collected and Western blot analyses were carried out essentially as described previously [28] using NuPAGE 4-12% Bis-Tris (reducing) and 3-8% Tris-Acetate (non-reducing) pre-cast gels (Invitrogen). Goat anti-human fibrinogen polyclonal antibodies (Covalab, France) and anti-goat IgG-HRP antibodies (Sigma, USA) were used.

### Reporter gene assay

The pGL4.10[luc2] (Promega) plasmid containing the *FGA* promoter region [29] was used as a template for two PCRs with the first primer pair around the promoter region and the second around the luciferase gene and polyA signal sequence (Table S1). Half of the first PCR was methylated using M.SssI and all PCR-products were digested with HindIII. Methylated or unmethylated *FGA* promoter fragments were ligated to the luciferase gene to create linear reporter genes with differentially methylated *FGA* promoters. Methylation was verified by digestion with the methylation-sensitive enzyme HpyCH4IV. DNA sequencing confirmed the expected sequences (for both ligation products an equal proportion contained a one nucleotide change at the ligation site). Luciferase assays were performed essentially as described [29] with 50ng of the promoter construct and 80ng (HuH7 cells) or 20ng (HEK293T cells) of Renilla luciferase control plasmid per well.

### Chromatin Immunoprecipitation (ChIP)

200-300 Zebrafish embryos were dissociated as described [30] to obtain a single cell suspension (per assay 8x10^6^cells). 20-30mg of frozen mouse liver and heart samples were minced. Cross linking, chromatin digestion and immunoprecipitation of mouse tissues and zebrafish single cell suspensions was performed using the SimpleChIP Plus Enzymatic Chromatin IP Kit (Cell Signaling Technology, Danvers,, USA). Per immunoprecipitation 2µg of Anti-Histone H3 (tri-methyl K4) (Abcam, Cambridge, United Kingdom) or control antibodies included in the kit against histone H3 and rabbit IgG were used. After DNA purification, enrichment was analyzed by qPCR using mouse *Rpl30* primers included in the kit and primers designed for the mouse or zebrafish fibrinogen genes and control regions (Table S1). The amount of immunoprecipitated DNA was calculated as signal relative to the total amount of input chromatin: 0.02*2^(C[T]2%Input sample – C[T]IP sample)^.

## Results

### Differential DNA methylation of the human fibrinogen locus in fibrinogen expressing and non-expressing cells

**Figure 1:**
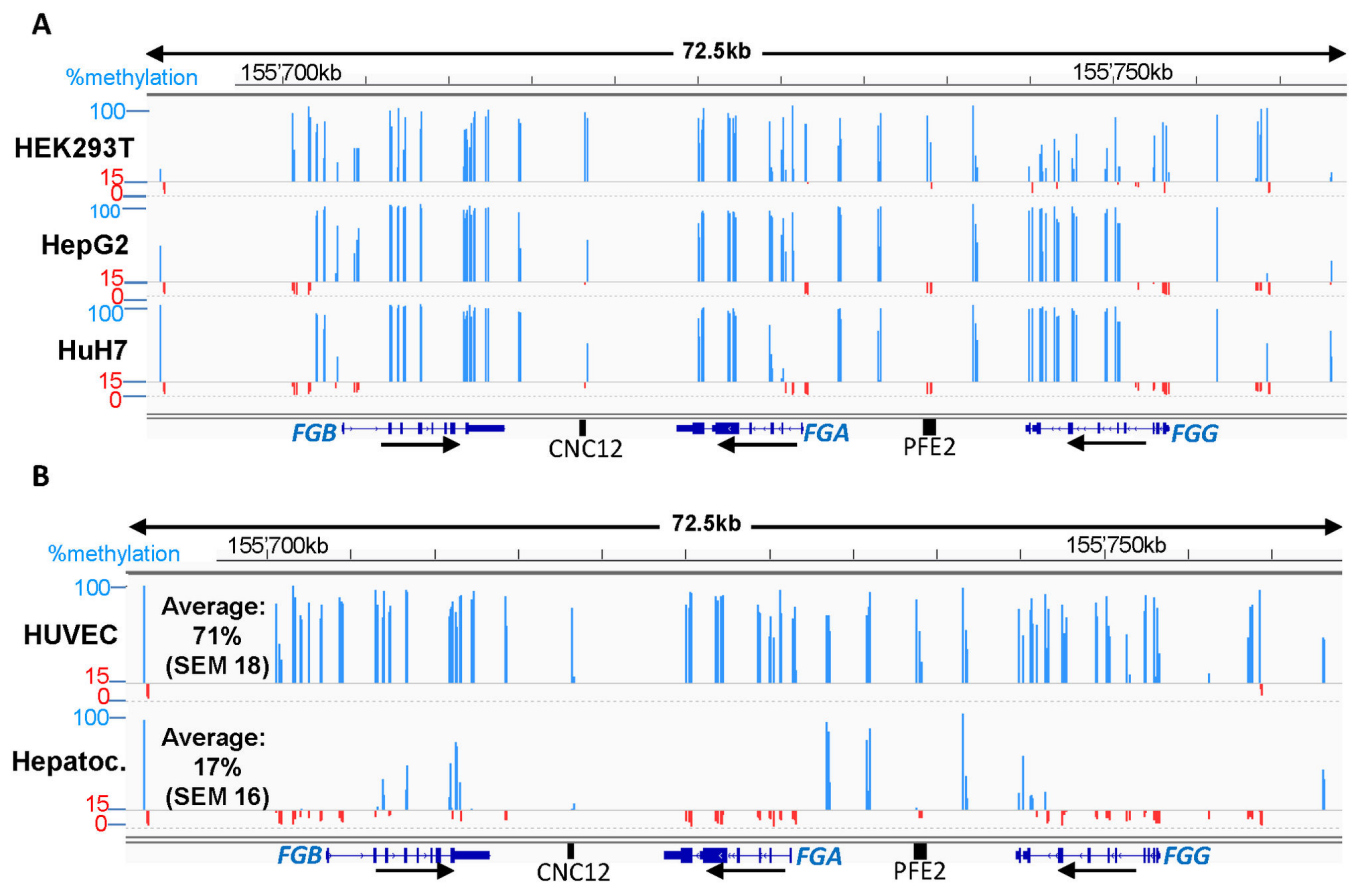
Differential methylation of the human fibrinogen locus in fibrinogen expressing and non-expressing cells. The reference genes (*FGB, FGA* and *FGG*) and the liver enhancers CNC12 and PFE2 are represented under each graph. Methylation percentages of the individual CpGs analyzed in this study are depicted as bars using the Integrative Genomics viewer IGV 2.1 [52] (NCBI36/hg18). A line is drawn arbitrarily at 15% methylation; values above are depicted as blue bars and values below as red bars. The peaks represent the mean methylation of 3 or 4 (hepatocytes) biological replicates. (A) Methylation profiles of human cell-lines: a non fibrinogen-expressing human cell line (HEK293T) and two fibrinogen-expressing hepatoma cell lines (HepG2 and HuH7). (B) Methylation profiles of human primary cells: non-fibrinogen expressing human umbilical vein endothelial cells (HUVEC) and fibrinogen expressing primary human hepatocytes. Average methylation percentages plus SEM across the whole locus are indicated.

We first compared the methylation profile of a 72.5kb region of human chromosome 4 containing the three fibrinogen genes in expressing and non-expressing human cells (Figure 1). Applying the criteria of Weber et al [31] for promoter classification the three fibrinogen promoters fall into the category of CpG-poor promoters. The 72.5kb fibrinogen locus contains 445 CpGs without clustering to satisfy the criteria applied to CpG islands (DNA-region ≥200bp; GC content ≥50% and observed/predicted CpGs ratio ≥0.6) [32]. Local enrichments of CpGs tend to occur in exons as compared to most intronic and intergenic regions. For a representative overview of CpG methylation in the locus, we used DNA methylation sequencing to analyze approximately 33% of all CpGs (144 CpGs) in the fibrinogen locus with a focus on promoter regions, gene bodies and the recently identified liver enhancers CNC12 and PFE2 [23,29].

HEK293T cells, that do not express fibrinogen, show an overall high degree of DNA methylation (average all analyzed CpGs ~60%, Figure 1A, Table S2), while the fibrinogen expressing cell lines, HuH7 and HepG2, show two patterns of methylation. CpGs in gene bodies and most intergenic regions are highly methylated (>70% methylation), whereas CpGs in all three promoter regions are mostly unmethylated (0-15% methylation, Figure 1A, Table S2). The same analysis was performed in human primary cells (Figure 1B). We found that non-fibrinogen expressing human umbilical vein endothelial cells (HUVECs) are even more methylated than HEK293T-cells (average methylation 71%, Table S2). In fibrinogen expressing hepatocytes CpGs across the fibrinogen locus generally show very low methylation (average methylation 17%) with little variability despite the heterogeneous background of the four samples (Table S2). Interestingly, the extremities of the 72.5kb region show five CpGs that are unmethylated (below 15%) in all cell types (fibrinogen expressing and non-expressing). These CpGs coincide with ChIP-seq peaks for CCCTC-binding factor (CTCF) in HepG2 cells [33]. The unmethylated sites common to all fibrinogen expressing cells are located upstream of the three transcription start sites (TSS), around the TSSs of *FGA* and *FGG*, as well as on the liver enhancers CNC12 and PFE2 [23,29]. These sites overlap ChIP sites for several transcription factors including HNF4α and C/EBP in HepG2 cells [34].

### CpG methylation in the FGA promoter reduces expression of a reporter gene

**Figure 2:**
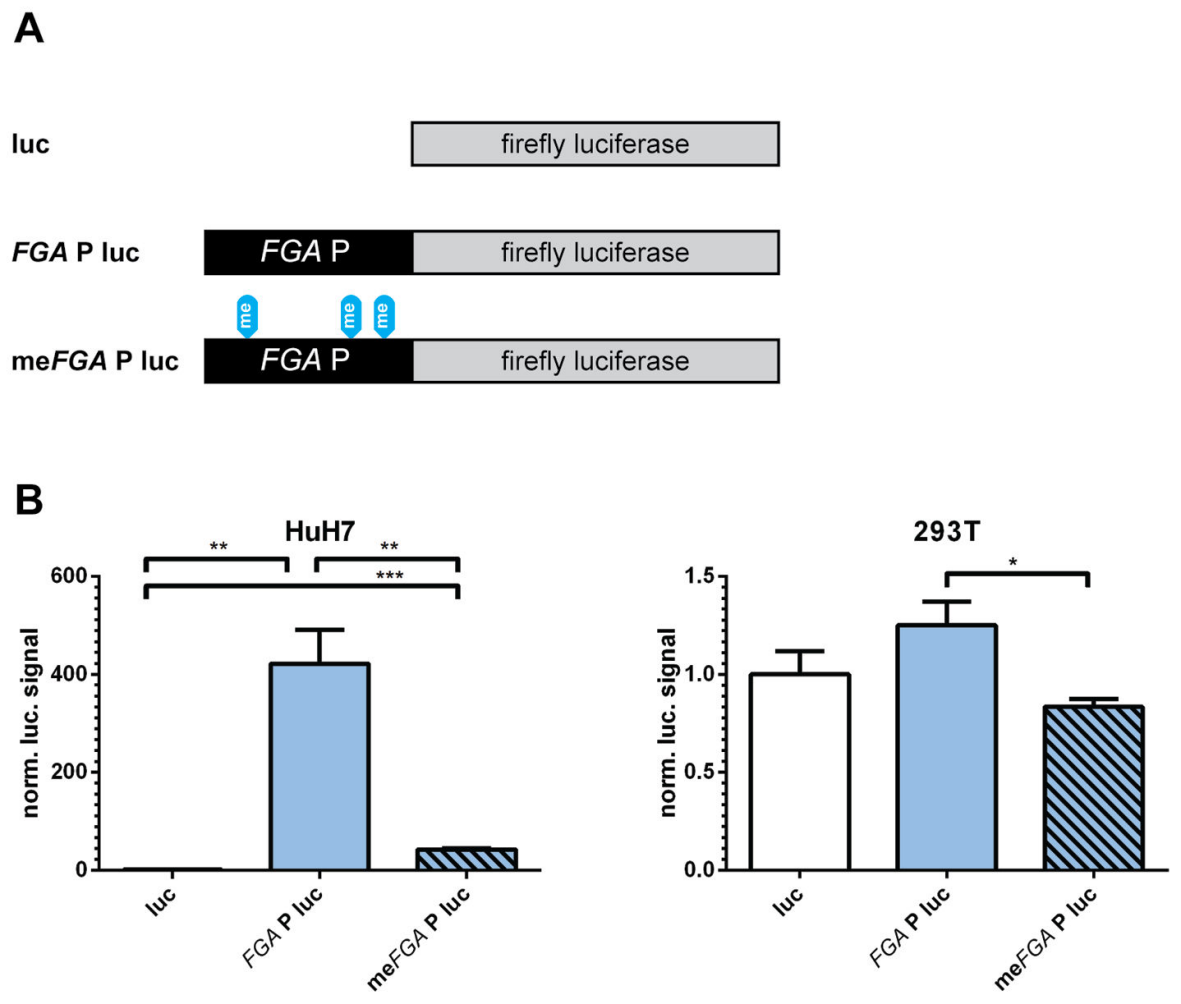
Methylation of the *FGA* promoter reduces expression of a reporter gene in fibrinogen expressing cells. (A) Linear constructs containing the differentially methylated *FGA* promoter (FGAP) and the firefly (ff) luciferase gene (luc). The differentially methylated promoter constructs were ligated to the luciferase gene and directly transfected without cloning steps. (B) Luciferase assays performed in fibrinogen expressing HuH7 cells and HEK293T control cells. Signals for the ligated fragments are normalized to the signal of the unligated luciferase gene. Bars show mean signals plus SEM, n=3. Statistical testing was performed using an unpaired *t* test (*P< .05, **P< .01, ***P< .001).

To determine whether differential CpG methylation has a direct effect on a CpG-poor fibrinogen promoter activity, we studied the effects of CpG methylation on the *FGA* promoter in a luciferase-based gene reporter assay. The promoter activity of this region has been described previously [29] and it contains three CpGs which are differentially methylated in fibrinogen expressing and non-expressing cells (Figure 1). To avoid confounding effects of a methylated plasmid backbone, we used differentially methylated linear *FGA* promoters ligated to an unmethylated firefly luciferase gene (Figure 2A). Compared to promoter-less luciferase gene activity, the unmethylated *FGA* promoter increased luciferase activity 400-fold in HuH7 cells, whereas the luciferase activity with the methylated *FGA* promoter was about ten-fold lower (Figure 2B). In HEK293T cells, the unmethylated *FGA* promoter does not significantly increase the background activity of the promoter-less luciferase; however, methylation of the *FGA* promoter still reduced luciferase activity around 1.5-fold.

### Interleukin-6 (IL-6) stimulation does not alter DNA methylation of the fibrinogen locus

As part of the acute phase reaction, fibrinogen gene expression increases during inflammation, particularly via IL-6-mediated signalling [35]. Interestingly, IL-6 has been shown to promote changes in DNA methylation during cancer development [36]. In order to test if IL-6-induced fibrinogen expression is associated with changes in DNA methylation at the fibrinogen locus, we treated cell lines with IL-6 and analyzed fibrinogen expression (by qPCR), fibrinogen protein secretion (by Western blotting) and DNA methylation. While IL-6 treatment was effective in increasing fibrinogen gene expression and fibrinogen protein production by HuH7 and HepG2 cells, no significant changes in DNA methylation were detected for individual CpGs or across distinct genomic regions in the fibrinogen locus (Figure S1). The same experiments were performed in HEK293T-cells, where IL-6 treatment neither activated fibrinogen gene expression nor influenced the DNA methylation level of the fibrinogen locus (data not shown).

### DNA methylation of the fibrinogen locus during mouse and zebrafish development

**Figure 3:**
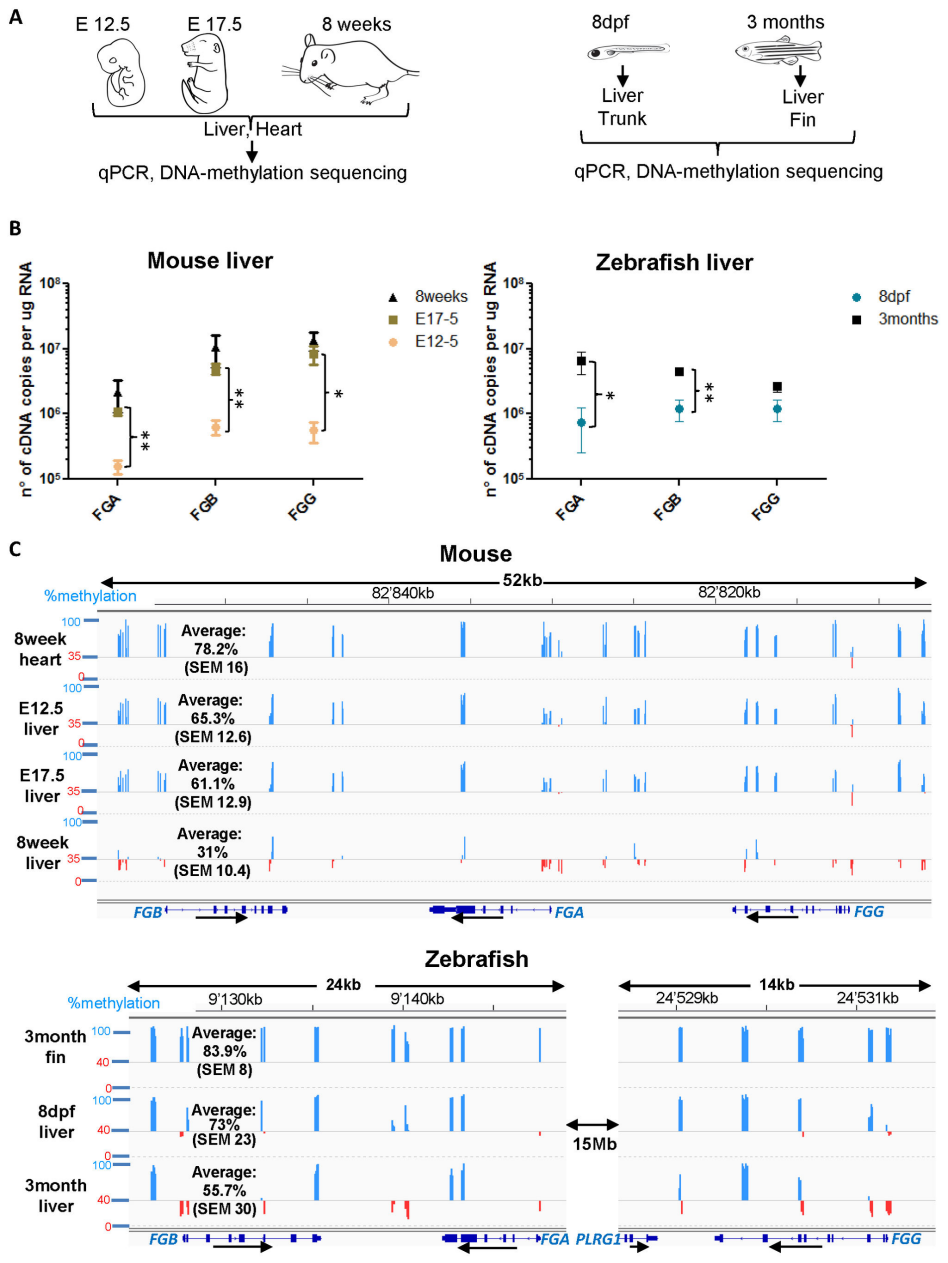
Changes in fibrinogen expression and fibrinogen DNA methylation during mouse and zebrafish development. (A) Experimental approach used to compare the changes in expression of the three fibrinogen genes with DNA methylation of the fibrinogen locus. The same mouse tissues were used to perform both experiments, except for E12.5 tissues, where two tissue samples were pooled per experiment (see Materials and Methods). For qPCR and DNA methylation sequencing on zebrafish tissues, the livers and caudal fins (for 3 month old females) or trunks (for 8dpf larvae) were dissected and DNA and RNA were extracted from the same samples. The experiments were performed with three biological replicates. (B) Absolute quantification of mouse and zebrafish fibrinogen RNA in the liver. The number of cDNA copies per µg RNA was determined by interpolation of the qPCR results on a standard curve of known concentrations of plasmids containing the respective cDNAs. The mean number of cDNA copies plus SEM are shown on a logarithmic scale, n=3. Statistical testing was performed using an unpaired *t* test (*P< .05, **P< .01). (C) Methylation percentages of individual CpGs analyzed are depicted as bars at their genome position using the Integrative Genomics viewer IGV [52] (for mouse: NCBI37/mm9, for zebrafish: Zv9/danRer7). A line is drawn at 35% methylation (mouse) or 40% (zebrafish); values above are depicted as blue bars and values below as red bars. Reference genes are shown below each graph and scales and genomic localization (mouse chromosome 3 reverse strand or zebrafish chromosome 1 forward strand) are shown above. Note that in zebrafish *fgg* is located ~15MB downstream of *fga*. The peaks represent the mean methylation of three biological replicates, the average methylation percentage plus SEM across the whole locus (for zebrafish the two loci shown) is given for each tissue.

We were interested in determining whether changes in DNA methylation were associated with changes in expression of the fibrinogen genes during liver development. Between mouse embryonic days 13 and 18 bi-potential hepatoblasts differentiate into biliary cells and hepatocytes [37], which in the latter is accompanied by a significant increase in expression of hepatic transcription factors [38]. We studied fibrinogen expression and fibrinogen locus DNA methylation at three stages of mouse liver development (Figure 3A): around the start of hepatocyte differentiation (E12.5), at a later stage where fetal hepatocytes are the predominant cell type in the liver (E17.5) and in the adult liver (8 weeks). Heart samples (non fibrinogen expressing controls) were analyzed at each developmental time point. We detected an almost ten-fold increase in hepatic expression of all three fibrinogen genes between E12.5 and E17.5 (Figure 3B). Between E17.5 and 8 week old livers a small, insignificant increase was detected. The main changes in basal fibrinogen expression therefore appear to occur during the differentiation of hepatoblasts into hepatocytes. To see if this ten-fold increase correlated with loss of DNA methylation, we performed DNA methylation sequencing on the same tissue samples. The mouse fibrinogen locus contains more CpGs than the human locus (560 CpGs in 45.4 kb), but as for the human locus, no CpG-islands are present and local enrichments can be detected in exons as compared to introns and intergenic regions.

We obtained CpG methylation percentages for approximately 20% of all CpGs present in the mouse fibrinogen locus. These did not include known polymorphic CpGs (Figure 3C, Figure S2, Table S2). CpG-methylation across the fibrinogen locus in non-expressing adult heart tissue is generally high with an average of 78%. The methylation of CpGs in the fibrinogen locus in adult mouse liver (8 weeks) is significantly lower with an average of 31%. As for human primary hepatocytes, low CpG-methylation stretches almost across the entire fibrinogen locus. However, unlike in human hepatocytes, we did not detect completely unmethylated CpGs. Given the mixed composition of liver tissue, this result is not surprising: since 70-80% of the liver consists of fibrinogen-expressing hepatocytes, up to 30% of CpG-methylation information is due to non-expressing biliary or endothelial cells. Mouse E12.5 livers show an average of 65.3% DNA methylation across the fibrinogen locus, which is only slightly higher than in E17.5 livers (average of 61.1%). The largest overall loss of fibrinogen locus methylation (down to 31% in 8 week livers) occurs later during liver development, after the greatest increase in basal fibrinogen gene expression detected in our study.

Similarly, we analyzed DNA methylation of the fibrinogen genes and fibrinogen expression during zebrafish development in embryonic liver (8 days post fertilization (dpf)) and adult liver (3 months). Since hepatic zebrafish fibrinogen expression is initiated between 24 and 48 hours post fertilization (hpf) [22], the larval liver at 8dpf is an intermediate stage between early embryonic liver and mature adult liver. After dissection of liver and control tissues (8dpf: trunk; 3-4 months: caudal fin), qPCR and DNA methylation sequencing was performed (Figure 3A). We found that liver *fga* expression increases approximately ten-fold between larval and adult stages, *fgb* expression increases approximately four-fold and *fgg* expression showed a non-significant two-fold increase (Figure 3B).

In contrast to human and mouse, the zebrafish fibrinogen genes are not arranged as a compact three gene cluster since *fgg* is located 15.4 Mb away from *fga/fgb* on chromosome 1 [22]. To assess the DNA methylation level of the three genes including flanking sequences harboring potential regulatory elements, we analyzed CpGs in the promoter and intronic regions as well as between *fgb* and *fga* and downstream of *fgg*. The zebrafish genomic regions encompassing *fgb/fga* and *fgg* (together 33.8kb) contain no CpG-islands. We analyzed 16% of all CpGs in the *fga/fgb* and *fgg* regions in the larval and adult tissues (Table S2). DNA methylation across the *fga/fgb* and *fgg* loci in non-fibrinogen expressing fins (Figure 3C) and larval trunks (Figure S2) is very high with an average of 83.9% (SEM 8). The adult liver shows approximately 30% less DNA methylation across the two loci (average of 55.3%, SEM 30) as compared to fins and trunks. As for the mouse, DNA methylation percentages reflect methylation status of the whole liver including cells other than hepatocytes. DNA methylation in zebrafish adult livers appears to follow two patterns: CpGs located in all three fibrinogen promoters, in the first half of *fgb* and *fgg* gene bodies, in the stretch between *fga* and *fgb* and downstream of *fgg* show low CpG methylation (<30%) while CpGs located at the 3’ end of the gene bodies and upstream of *fgb* are highly methylated (above 70%). In the larval liver the average CpG methylation was 73% with a similar distribution to that of the adult liver. Thus, the loss of DNA methylation during zebrafish liver development partially correlates with changes in fibrinogen expression: the larval liver shows an intermediate DNA methylation of all fibrinogen genes as compared to adult liver and control tissues, whereas only *fga* and *fgb* expression increase significantly between larval and adult livers.

### H3K4me3 at the fibrinogen locus during mouse and zebrafish development

**Figure 4:**
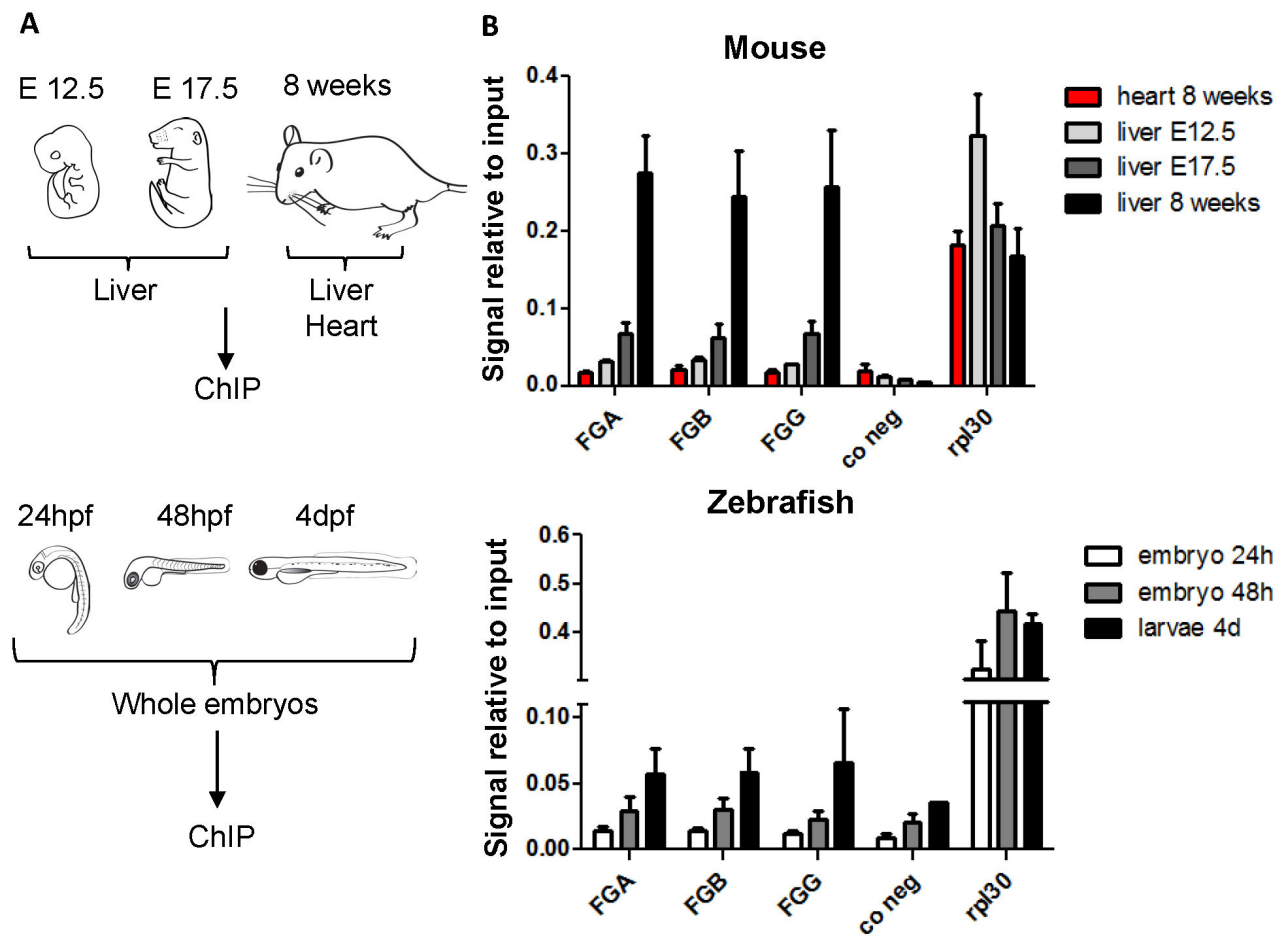
Active chromatin mark H3K4me3 on the fibrinogen promoters during mouse and zebrafish development. (A) Overview of the samples used for Chromatin immunoprecipitation (ChIP). For mouse experiments, the 8 week tissues are the same as those used for qPCR and DNA methylation sequencing, the E17.5 and E12.5 mice are from the same crossing as the embryos used for Figure 3. For zebrafish experiments, ChIP was performed on whole embryos at different stages. All experiments were performed with three biological replicates, except for mouse E12.5 liver, where two pools of five livers were used. (B) Enrichment of H3K4me3 on mouse and zebrafish fibrinogen promoters and control regions. After ChIP on tissues or whole zebrafish embryos the signal relative to the total input was determined by qPCR, using primers situated around the transcription start site or in the first intron of each fibrinogen gene (Table S1). Primers binding to the first intron of the ubiquitously expressed *Rpl30* gene serve as a positive control for enrichment. As a negative control for the H3K4me3 enrichment, gene free regions on chromosome 12 (mouse) and 14 (zebrafish) were chosen. The mean enrichment plus SEM is shown. Anti-rabbit IgG was used as a negative control in the ChIP experiments and the signals per total input were <0.0007 for all genes examined.

Trimethylation of lysine 4 on histone H3 is a mark of active promoters and its presence correlates strongly with hypomethylated CpGs [19]. In the fibrinogen locus, hypomethylated CpGs in fibrinogen promoters of expressing human cells or mouse livers overlap with known H3K4me3 ChIP-seq peaks in HepG2 cells [34] or mouse livers [39]. In order to analyze this association more deeply we determined whether trimethylation of H3K4 in the fibrinogen promoters changes during development. We performed chromatin immunoprecipitation (ChIP) using an antibody against H3K4me3 on mouse tissues (Figure 4A). All three fibrinogen promoters give the strongest signal in adult mouse liver (Figure 4B) with values around 0.25 similar to those obtained for the positive control *Rpl30* (0.2-0.3). During mouse liver development, the signal increases two-fold between E12.5 and E17.5 and more than three-fold between E17.5 and adults. H3K4me3 signals on fibrinogen promoters appear therefore to increase during mouse liver development at the same time as the surge in basal fibrinogen expression and before loss of DNA methylation.

In adult zebrafish, a liver specific enrichment of H3K4me3 in all three fibrinogen promoters as compared to a negative gene-free region could be detected (data not shown). Unfortunately, a direct comparison to enrichment in 8 dpf larval liver was hindered by the limited liver material obtained by dissections which was sufficient for qPCR and DNA methylation sequencing (Figure 3) but not for reproducible ChIP. Instead, we asked whether we can detect H3K4me3 enrichment on the fibrinogen promoters during zebrafish embryonic development and if this enrichment changes during the initiation of hepatic fibrinogen expression between 24 and 48 hpf [22]. Therefore, we performed ChIP on whole embryos at 24 hpf, 48 hpf and 4 dpf (Figure 4A). As depicted in Figure 4B, the strongest signals for the fibrinogen promoters were obtained for the 4 dpf larvae (0.06 for *fga, fgb*; 0.07 for *fgg*); even though clearly below *rpl30* signals (0.3-0.4), they are still significantly above values for the negative control (gene-free region on chromosome 14, signal 0.03). Fibrinogen promoter signals for the 24 and 48 hpf embryos are around 0.01 and 0.03 respectively, in the range of negative control signals. In summary, we detected H3K4me3 only in 4dpf embryos, after the initiation of hepatic fibrinogen expression. However, we cannot exclude that H3K4me3 is present but not detectable in earlier stages because of relative low abundance of fibrinogen expressing cells in 24 and 48 hpf embryos, or due to limited sensitivity of the ChIP-method. 

## Discussion

This study presents the global CpG methylation status of the fibrinogen gene cluster of three species and relates it to fibrinogen expression and histone modification. We found that human cells show differentially methylated regions in the fibrinogen locus correlating with fibrinogen expression. In non-fibrinogen expressing cells, CpGs are in general highly methylated. Notable exceptions are several CpGs at the ends of the locus, which are unmethylated in all cells analyzed. These sites overlap CTCF ChIP-seq peaks [33] and could be implicated in setting up a higher order chromosome structure of the fibrinogen locus since active CTCF binding correlates with low CpG-methylation at the binding sites [40]. Fibrinogen-expressing human hepatoma cell lines show a high level of methylation at the fibrinogen locus which drops sharply around the promoter and enhancer regions. These results are in concordance with studies identifying unmethylated, CpG-poor promoters in tissues where the genes are expressed [17,41,42] and low DNA methylation levels at active enhancers [43]. Interestingly, the regions of low DNA methylation in primary human hepatocytes cover almost the whole fibrinogen locus including gene bodies. This observation is in line with a previous study [44] showing low levels of gene body methylation for highly expressed genes. Since hepatoma development and culture conditions have been associated with changes in the cell methylome [45,46], this may explain the differences we observe between cell lines and primary cells.

Previous studies indicate a repressive potential for DNA methylation in CpG-poor promoters [18], and our *in vitro* data using a luciferase gene reporter assay supports a repressive role for DNA methylation of the CpG-poor *FGA*-promoter. However it is still not clear if loss of DNA methylation is implicated in gene activation [12]. We determined whether loss of DNA methylation at the fibrinogen locus is associated with an induced increase in fibrinogen gene expression. We analyzed DNA methylation after treatment with IL-6, a well-known stimulator of fibrinogen expression [35] and a cytokine associated with changes in DNA methylation levels in cancer [36]. Under our experimental conditions we could not find any relationship between IL-6 induced fibrinogen expression and changes in DNA methylation level at the fibrinogen locus.

During hematopoietic differentiation, Calvanese et al [17] detected a correlation between loss of methylation and active expression of mainly CpG-poor promoter genes. Our analyses of mouse and zebrafish tissues show a loss of DNA methylation around the fibrinogen genes during liver development. Recent whole genome studies indicate abundance of transcription factors as the main determinant of DNA methylation status [47] and our results seem to point in the same direction. We detect a ten-fold increase in basal fibrinogen expression in mouse liver between E12.5 and E17.5, a developmental period characterized by increased expression of major hepatic transcription factors [38], whereas the most significant loss of DNA methylation occurs after E17.5. The analysis of DNA methylation in zebrafish larval and adult liver also shows a gradual loss of DNA methylation around the fibrinogen genes. *fgb*- and *fga*-expression increase four- and ten-fold respectively between larval and adult liver while *fgg*-expression levels increase non-significantly. During the same developmental time frame, DNA methylation levels of all three fibrinogen gene promoters decrease by approximately 20%.

DNA methylation in association with histone marks, such as unmodified H3K4, can indicate repressed chromatin. However, the direct functional impact of these marks on transcription is not fully understood [48]. Our data suggest that changes in fibrinogen expression could be a driver for trimethylation of H3K4 on the fibrinogen locus, since we detect the strongest enrichment of H3K4me3 at the fibrinogen promoters after the ten-fold increase in mouse fibrinogen expression. In zebrafish, we detected H3K4me3 only after initiation of hepatic transcription in zebrafish. However, further experiments should clarify if the histone mark is indeed preceded by transcriptional activation of the fibrinogen genes or if it is not detected due to technical limitations. P300 ChIP-seq peaks have been detected in HepG2 cells across several sites at the human fibrinogen locus [34] and might be connecting liver specific transcription with histone modifications since P300 is necessary for both liver specification and histone acetylation at liver-specific regulatory elements [49]. Interestingly, we detected a significant two-fold enrichment of H3K4me3 in the mouse fibrinogen promoters between E12.5 and E17.5, which precedes the loss of DNA methylation around the fibrinogen genes. These results indicate a potential temporal order for changes in these two epigenetic marks on the fibrinogen locus; the H3K4me3 mark precedes the loss of CpG methylation. CpG methylation is then gradually lost once the histone mark is established, perhaps due to the reduced activity of a DNA-methyltransferase, such as DNMT1, which is preferentially stabilized at unmodified H3K4. The loss of CpG methylation could also require cell divisions without methylation of new DNA molecules in daughter cells [50]. 

**Figure 5:**
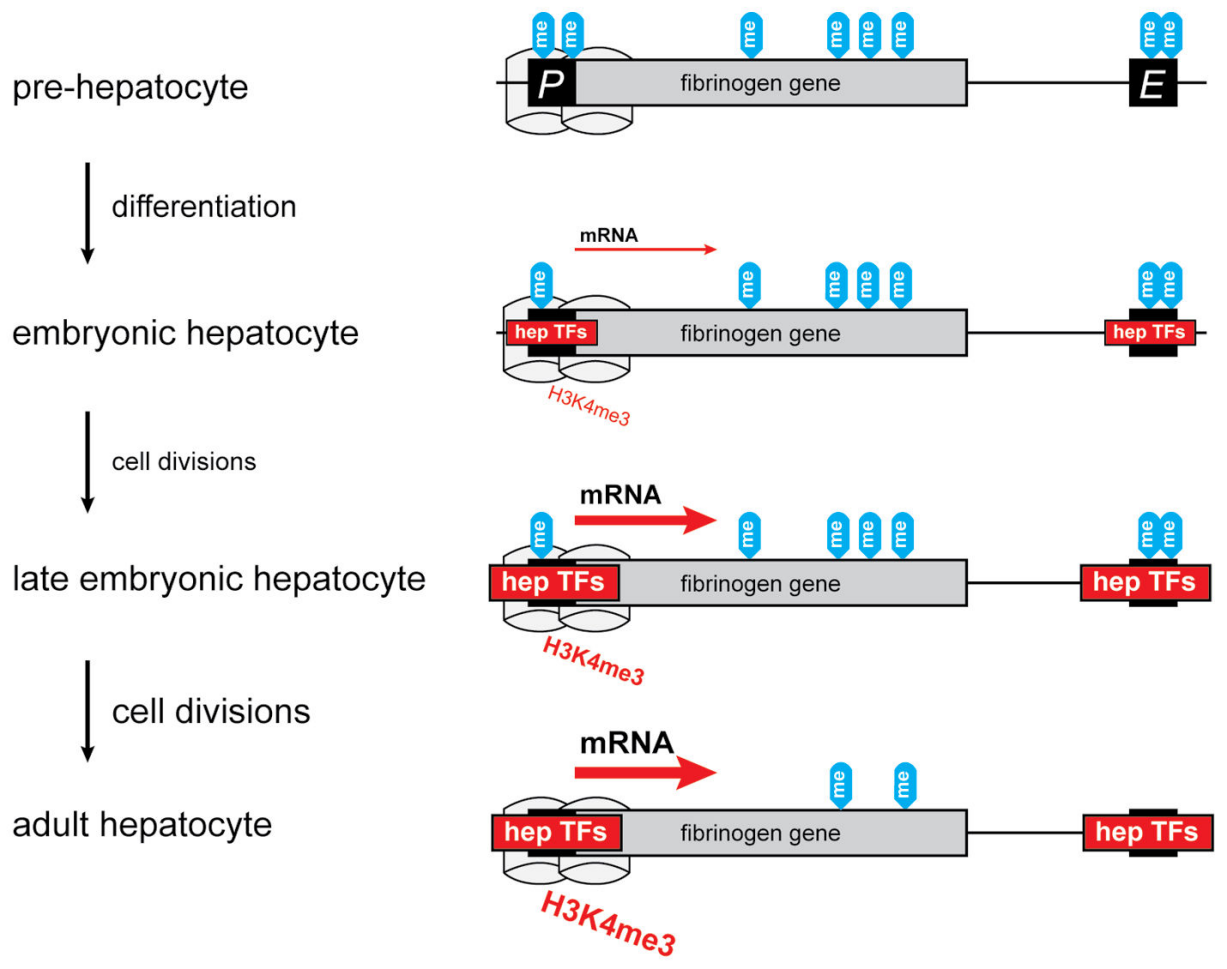
A model of changes in epigenetic marks and expression of fibrinogen genes during development. A single fibrinogen gene is shown here to represent the three vertebrate fibrinogen chain genes and the model represents an average situation in a population of cells. An upstream promoter (*P*) and an intergenic liver enhancer (*E*) sequence are also represented. Prior to hepatocyte differentiation, CpGs in the fibrinogen genes are methylated (“me”) both in regulatory sequences and across the gene bodies. Embryonic hepatocytes show similar CpG methylation patterns, but with the expression of hepatocyte transcription factors (hep TFs) regulatory sequences are employed and fibrinogen mRNA expression is measurable. The H3K4me3 histone mark is also detectable near the gene promoters (grey cylinders). In later embryonic hepatocytes, expression of fibrinogen is clearly increased (red arrow), the H3K4me3 mark is more abundant at promoters and CpG methylation is moderately reduced, perhaps as a result of the loss of repressive CpG methylation upon cell division. In adult hepatocytes expression is marginally higher still, but CpG methylation is markedly reduced, in gene bodies and regulatory elements.

Our *in vitro* results suggest a contribution for DNA methylation in reducing the activity of the CpG-poor *FGA*-promoter. Methyl-CpG-binding proteins (MBPs) may play a role in tissue-specific silencing of the fibrinogen genes since they recognize methylated cytosines, attract transcriptional co-repressor molecules and link DNA methylation and chromatin remodeling by binding chromatin modifiers [51]. Determining whether MBPs are bound to methylated fibrinogen promoters and if this binding is modified by hepatic transcription factors would link the repressive potential of DNA methylation to our observation that DNA methylation is lost after the increase of basal fibrinogen expression during liver development. While interplay between transcription factors, histone modifiers and DNA-methyltransferases has been shown [19,47], our results on the fibrinogen locus suggest a hierarchy of events. The increase in basal hepatic fibrinogen expression is the primary event, followed by trimethylation of H3K4me3 and loss of DNA methylation. Thus we propose a model (Figure 5) in which DNA methylation of the fibrinogen locus is implicated in the maintenance of a silent state which is reshaped by active transcription and histone modification during tissue differentiation.

## Supporting Information

Figure S1 **IL-6 treatment of HuH7 and HepG2 cells.** (A) Quantitative RT-PCR: Relative expression of the three fibrinogen genes in HuH7 and HepG2 cells after treatment with 100ng/ml IL-6 for the indicated time. Each time point was normalized to the untreated control cells. The error bars represent the SEM of the relative expression of three biological replicates.
(B) Western blot analysis of conditioned media from HuH7 and HepG2 cells under non-reducing and reducing conditions. FG ctrl: purified fibrinogen control; co: untreated control cells; IL-6: cells treated with 100ng/ml IL-6 for 24h. (C) Box-plots of the DNA methylation percentages for the promoter and gene bodies/intergenic group. To determine if IL-6 had a small effect on DNA methylation across a whole region, we grouped the CpGs according to their methylation status in untreated control cells. The average methylation was calculated across a “promoter” group consisting of CpG regions with low methylation percentage (≥ 3 CpGs with ≤ 30% DNA methylation) and a “gene bodies/intergenic” group (> 30% DNA methylation). The top and bottom of the boxes are the 75th and 25th percentile and the whiskers represent the 10-90th percentile. The DNA methylation profile of the untreated control cells is compared to the mean methylation profile of cells treated with 100ng/ml IL-6 for 6, 16 and 24h.
(TIFF)

Figure S2 **Methylation profile of the fibrinogen locus in control tissues.** The mean methylation percentages of individual CpGs for non-expressing control tissues are depicted as bars at their genome position using the Integrative Genomics viewer IGV (for mouse: NCBI37/mm9, for zebrafish: Zv9/danRer7). A midline is drawn at 35% methylation (mouse) or 40% (zebrafish); values above are depicted as blue bars and values below as red bars. At the bottom of the graphs the reference genes are shown and the scales at the top of the graphs show the location on mouse chromosome 3 (reverse strand) or on zebrafish chromosome 1 (forward strand). The average methylation percentage plus SEM across the whole locus (for zebrafish two loci) is given. (A) Methylation profile of mouse embryonic heart. (B) Methylation profile of zebrafish larval trunk.
(TIFF)

Table S1 **List of primers used for this study.**
(XLSX)

Table S2 **Mean methylation percentages and SEM per CpG for all cells and tissues.**
(XLSX)
