# Supplementary figures and images for: DNA Methylation Profiling of the Fibrinogen Gene Landscape in Human Cells and during Mouse and Zebrafish Development
Source: PLoS One. 2013 Aug 21;8(8):e73089. doi: 10.1371/journal.pone.0073089 (PMC3749180; doi:10.1371/journal.pone.0073089)

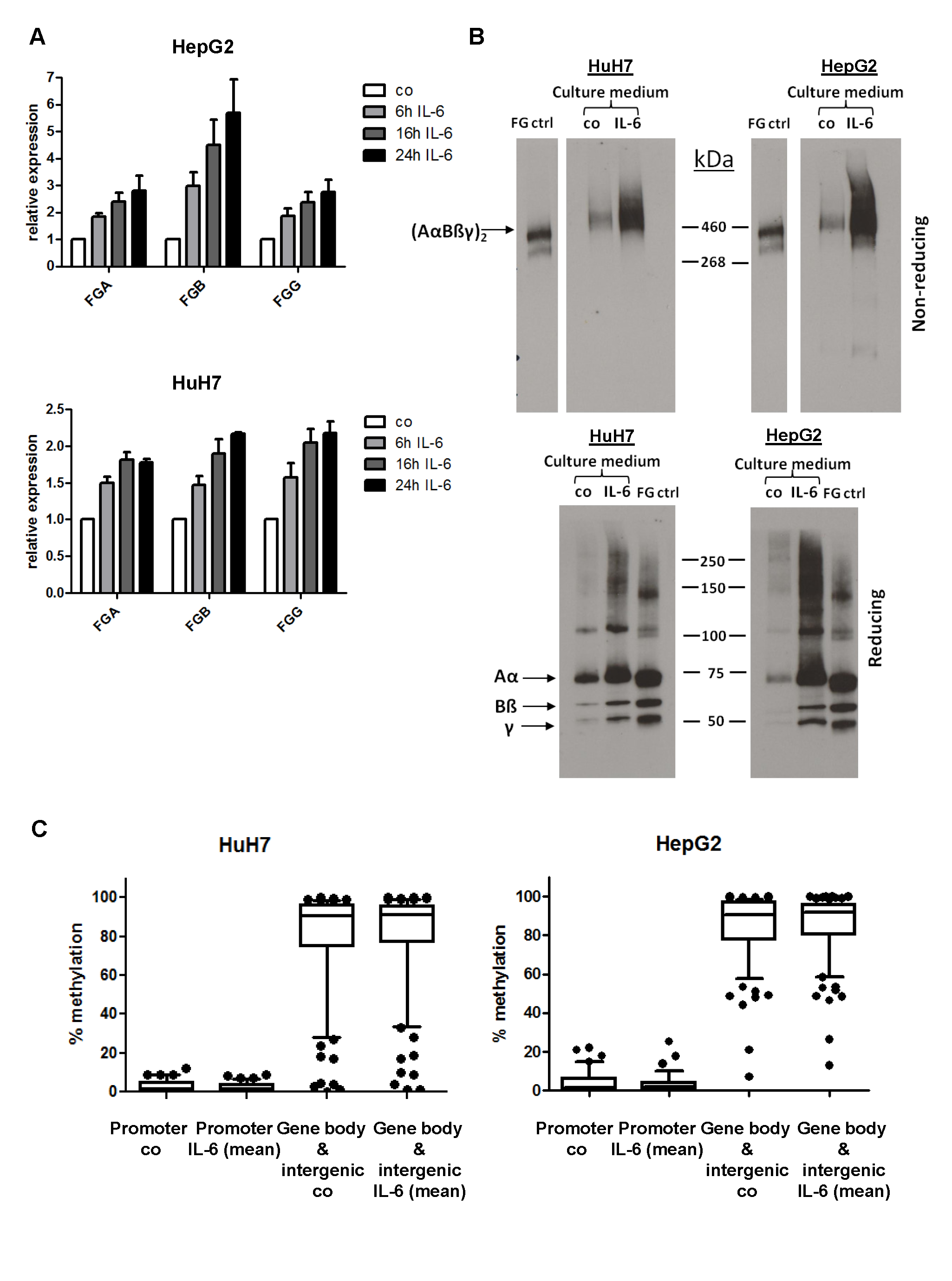

Supplement: Figure S1 — IL-6 treatment of HuH7 and HepG2 cells. (A) Quantitative RT-PCR: Relative expression of the three fibrinogen genes in HuH7 and HepG2 cells after treatment with 100ng/ml IL-6 for the indicated time. Each time point was normalized to the untreated control cells. The error bars represent the SEM of the relative expression of three biological replicates. (B) Western blot analysis of conditioned media from HuH7 and HepG2 cells under non-reducing and reducing conditions. FG ctrl: purified fibrinogen control; co: untreated control cells; IL-6: cells treated with 100ng/ml IL-6 for 24h. (C) Box-plots of the DNA methylation percentages for the promoter and gene bodies/intergenic group. To determine if IL-6 had a small effect on DNA methylation across a whole region, we grouped the CpGs according to their methylation status in untreated control cells. The average methylation was calculated across a “promoter” group consisting of CpG regions with low methylation percentage (≥ 3 CpGs with ≤ 30% DNA methylation) and a “gene bodies/intergenic” group (> 30% DNA methylation). The top and bottom of the boxes are the 75th and 25th percentile and the whiskers represent the 10-90th percentile. The DNA methylation profile of the untreated control cells is compared to the mean methylation profile of cells treated with 100ng/ml IL-6 for 6, 16 and 24h. (TIFF) [file pone.0073089.s001.tiff]

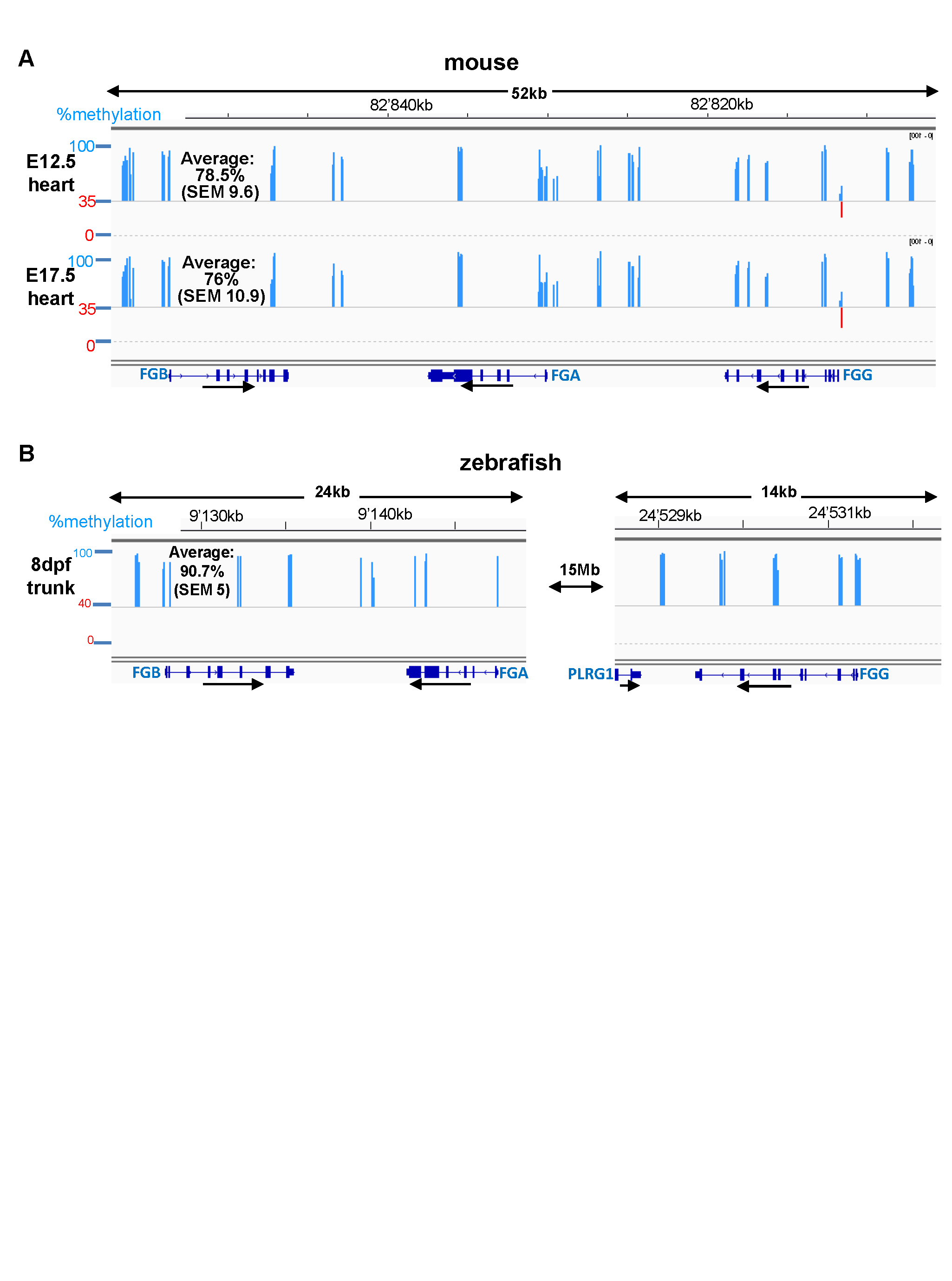

Supplement: Figure S2 — Methylation profile of the fibrinogen locus in control tissues. The mean methylation percentages of individual CpGs for non-expressing control tissues are depicted as bars at their genome position using the Integrative Genomics viewer IGV (for mouse: NCBI37/mm9, for zebrafish: Zv9/danRer7). A midline is drawn at 35% methylation (mouse) or 40% (zebrafish); values above are depicted as blue bars and values below as red bars. At the bottom of the graphs the reference genes are shown and the scales at the top of the graphs show the location on mouse chromosome 3 (reverse strand) or on zebrafish chromosome 1 (forward strand). The average methylation percentage plus SEM across the whole locus (for zebrafish two loci) is given. (A) Methylation profile of mouse embryonic heart. (B) Methylation profile of zebrafish larval trunk. (TIFF) [file pone.0073089.s002.tiff]
